# RMechDB: A Public Database of Elementary Radical Reaction Steps

**DOI:** 10.1021/acs.jcim.2c01359

**Published:** 2023-02-17

**Authors:** Mohammadamin Tavakoli, Yin Ting T. Chiu, Pierre Baldi, Ann Marie Carlton, David Van Vranken

**Affiliations:** † Department of Computer Science, University of California, Irvine, Irvine, California 92697, United States; ‡ Department of Chemistry, University of California, Irvine, Irvine, California 92697, United States

## Abstract

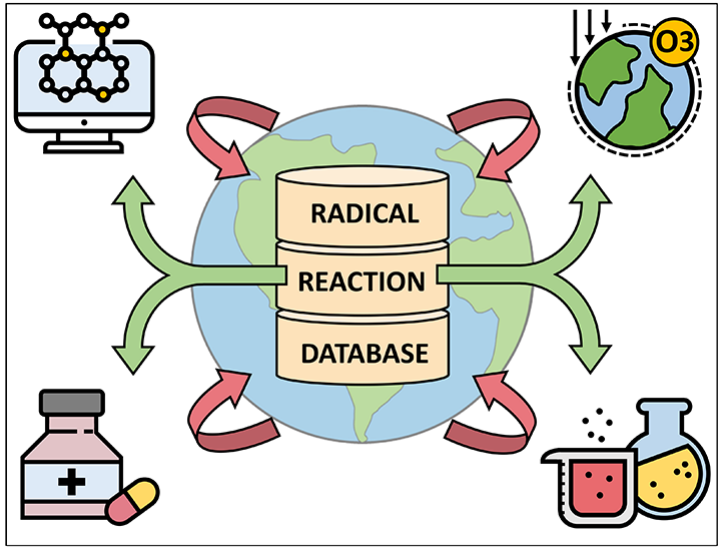

We introduce RMechDB, an open-access platform for aggregating, curating, and distributing reliable data about elementary radical reaction steps for computational radical reaction modeling and prediction. RMechDB contains over 5,300 elementary radical reaction steps, each with a single transition state at or around room temperature. These elementary step reactions are manually curated plausible arrow-pushing steps for organic radical reactions. The steps were taken from a variety of sources. Over 2,000 mechanistic steps were extracted from textbooks and/or constructed from research publications. Another 3,000 were taken from gas-phase atmospheric reactions of isoprene and other organic molecules on the MCM (Master Chemical Mechanism) Web site. Reactions are encoded in the SMIRKS format with accurate atom mapping and annotations for arrow-pushing mechanisms. At its core, RMechDB consists of a database schema with an online interactive search interface and a request portal for downloading the raw form of elementary step reactions with their metadata. It also offers an interface for submitting new reactions to RMechDB and expanding the data set through community contributions. Although there are several applications for RMechDB, it is primarily designed as a central platform of radical elementary steps with a unified and structured representation. We believe that this open access to this data and platform enables the extension of data-driven models for chemical reaction predictions and other chemoinformatics predictive tasks.

## Introduction

A free radical is a chemical compound (e.g., atom, molecule) with at least one-half-occupied orbital. The presence of the half-occupied orbitals makes a radical compound highly reactive. Because of this high reactivity, free radicals have the potential to both serve as powerful chemical tools and be extremely harmful contaminants. Chemical reactions involving a free radical are radical reactions that are an essential part of synthetic, biochemical, atmospheric, and plasma chemistry.^1−3^ For instance, the climate crisis has dramatically altered fire activity worldwide. Wildland fires are increasing in frequency, duration, intensity, and size. The chemistry of flames is dominated by radical reactions, and the chemical composition of fire smoke changes during atmospheric transport. This so-called “aging” of smoke is poorly understood but known to be largely driven by free radical processes.^1,4,5^ As another example from the pharmaceutical industry, the composition of drug formulations changes gradually upon storage. As a result, all drug companies are required to study those changes through forced degradation studies under several conditions, including photochemical and oxidative conditions, which mostly involve radical reactions.^6,7^ Thus, it is of great importance to study the chemistry of radical reactions and their outcomes.

During the past few years, data-driven methods such as deep learning have provided new powerful tools for addressing chemoinformatics problems.^8,9,11−14^ Due to important applications ranging from automated drug discovery to computer-aided synthetic chemistry, there has been an increasing interest in developing deep learning models to predict the outcome of chemical reactions.^15−19^ While the deep learning models have been evolving in sophistication and complexity, a major stumbling block has remained the lack of comprehensive, standard, and public, reaction data.^20^ The majority of recently developed models is being trained using the data set of chemical transformations from the US Patent office,^21^ as well as a few other smaller data sets.^10,22,23^ These data sets are spread across different platforms without unified and structured representations and metadata. Additionally, they suffer from significant limitations in terms of overall size, chemistry coverage and balance, and lack of metadata, atom mapping, reactant or product balance, and elementary reaction step information. For instance, the USPTO data set of chemical reactions restrictively represents chemical reactions in the form of overall transformations, most of which lead to one single major product. It contains little information about underlying mechanisms and about key intermediates and side products. Furthermore, radical reactions are hard to extract and appear to be underrepresented. On the other hand, radical reactions often proceed through a complex series of chemical steps and highly branched mechanistic pathways. Developing an accurate machine learning model for predictive tasks on radical reactions (e.g., predicting the outcome of radical reactions) requires a training data set of purely radical reactions with information about the mechanistic pathways and intermediate products. To overcome the above limitations and provide a source of data for radical reactions with their unique natural characteristics, we developed RMechDB as a central platform for aggregating, curating, and distributing elementary step radical reactions. RMechDB is designed as an extendable database schema, capable of hosting huge sources of radical reactions in the form of elementary steps. RMechDB is publicly available in the form of an online web server with interactive interfaces where users can search, download, and upload elementary step radical reactions. The initial version of the RMechDB data set consists of over 5300 manually curated radical reactions and is accessible through the DeepRXN Web site at https://deeprxn.ics.uci.edu/rmechdb.

## Mechanistic Pathways vs Overall Transformations

**Figure 1:**
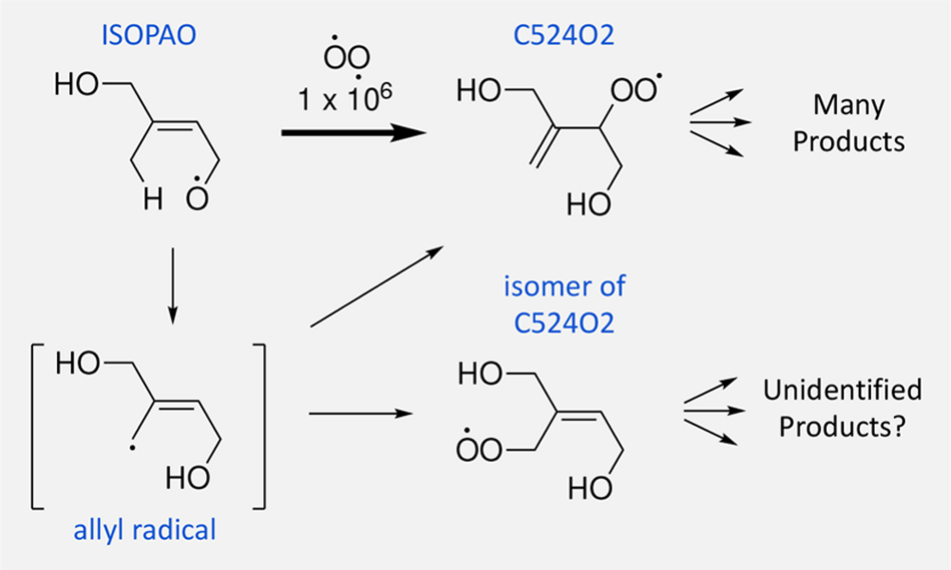
Missing steps and intermediates prevent identification of products. The formation of an allyl radical was not depicted for the transformation of ISOPAO to C524O2 in the MCM. It is not clear why the missing allyl radical intermediate would not also generate an isomer of C524O2 and account for more downstream products.

The term reaction can be ambiguous and is most commonly used to describe either 1) a chemical transformation with reactants, products, chemical conditions and yields or 2) a single step in an arrow-pushing mechanistic pathway. Therefore, in this work, instead of using the vague term of “reaction”, we use the more specific terms of transformation and elementary step to refer to the definitions above, respectively. Every mechanistic pathway can be decomposed into a series of discrete elementary steps, each with a single transition state.^24,25^ In several aspects, it is advantageous to show every step in a mechanistic pathway. First, when all the steps in a pathway are elementary, there is no chance of missing key intermediates that give rise to competing pathways during chemical transformation. This becomes extremely important with the presence of free radicals as radical transformations often proceed through a complex series of chemical steps and highly branched mechanistic pathways. For example, when the transformation of ISOPAO to C524O2 is depicted as a one-step process, it misses the potential for the allyl radical intermediate to form an isomeric peroxy radical and downstream products (Figure 1). The second advantage to mechanistic pathways based on elementary reaction steps is that they can be described using curved half arrows that correspond to the interaction of singly occupied molecular orbitals with a HOMO and/or LUMO.^26^ The curly arrows, also known as electron flow specifications or arrow-pushing mechanisms, are depicting the interaction between molecular orbitals. This representation of elementary steps is highly informative, and when elementary steps are chained together, an interpretation of the corresponding transformation can readily be derived. This becomes even more important specifically for deep learning approaches to reaction product prediction for at least three reasons. First, the prediction of mechanistic pathways leads to predictions that are interpretable. Interpretability is an important consideration in machine learning, especially for so-called “black-box” approaches such as deep learning.^27,28^ Second, when machine learning models operate at the level of elementary steps, the balance between reactants and products is always preserved together with the underlying atom mapping. Maintaining the balance through a chain of reactions can be extremely important in the study of retrosynthesis pathways. And third, by considering the pathways, all intermediary and final products can be accounted for, which is an important consideration in synthetic chemistry applications.

Given the crucial advantages of representing chemical reactions in the form of mechanistic pathways, it is highly beneficial to synthesize a data set of elementary radical steps. Such data sets can facilitate the training and development of deep learning models that are able to automate complex predictive tasks in radical chemistry.

## Approaches to Chemical Reaction Modeling and Predictions

An open-source, publicly available database of pedagogical elementary reaction steps will facilitate training and development of tools for automating chemoinformatics tasks such as the prediction of reaction mechanisms. There are two common approaches to the prediction of stepwise mechanisms of organic transformations using databases of elementary reaction steps. The quantitative approach uses a database of kinetic and thermodynamic parameters to accurately predict the products of the reactions and the pathways by which they form. This approach, as it is used in refs (29 and 30), is not restricted to elementary reaction mechanisms, but it does require kinetic parameters. The approach is best applied to cases where the product structures are known, but the abundances are not known. The qualitative approach such as used in refs (10, 15, and 31) uses a database of diverse plausible (fast at or below 100 °C) mechanistic steps, to match chemical structures (and mechanistic pathways) to mysterious, unknown, or not structurally characterized analytes in readily available spectra or chromatograms. This approach is best applied when the abundance is known, but the chemical structure is unknown. The chemical structure can provide powerful insight into biological effects, phase partitioning, and reactivity under changing reaction conditions. Public databases of mechanistic steps will empower the use of machine learning to create tools that assign chemical structures and mechanisms to products of environmental, synthetic, and environmental transformations of organic compounds.

## Existing Data Sets of Elementary Reaction Steps

There are several large commercial databases of organic transformations such as REAXYS, SciFinder, and very few open-access databases such as the Open Reaction Database (ORD).^32^ Those databases are composed of recipes that describe reactants, conditions, yields, and a list of products that rarely sums to 100%. The proprietary REAXYS database currently has over 57 million transformations. The SciFinder Scholar database has over 126 million transformations, which includes sequential reactions. Organic transformations were mined from US Patents from 1976 to 2016 and are publicly available. The growing ORD already gathers about 2 million chemical transformations from other available sources.^32^ These databases of chemical transformations allow synthetic organic chemists, or systems trained with machine learning,^33^ to plan out synthetic routes composed of sequential laboratory experiments, but the data do not reveal the underlying mechanisms of any individual transformations. Databases of transformations are not new, and neither is the application of AI to the planning of synthetic routes. Why is there no database of elementary arrow-pushing reaction steps? Sadly, when curved arrows were first introduced in 1922,^34,35^ the connection between curved arrows, frontier orbitals, and transition states was not recognized, so there was no incentive to apply them solely to elementary mechanistic steps. As a result, curved arrow mechanisms and half arrow radical mechanisms have been used inconsistently, throughout the organic chemistry literature, and are rendered in graphical forms that are not easily recoverable through data mining. Reaction Mechanism Generator (RMG) supports the only existing database of elementary mechanistic reaction steps. RMG predicts mechanistic pathways through a quantitative approach, using thermochemical and kinetic parameters to model species concentrations and rates for each step.^29^ RMG is supported by a searchable database, consisting of 98 families of reaction types.^29^ Almost half (40/98) of the reaction families in the current RMG database involve radicals. About a fourth of the reaction families supported by RMG do not correspond to elementary reaction steps at or around room temperature (e.g., unimolecular keto–enol tautomerization). Most of the mechanistic steps and kinetic data were developed to support high-temperature processes up to 2000 K, and many of the steps would be implausibly slow at room temperature. For example, the kinetic parameters for homolysis of a CH_3_ group from isoprene would proceed with a half-life of over 10^42^ years. Many of the steps that proceed through a single transition state at high temperatures (e.g., over 1500 K) would involve more than one mechanistic step at room temperature.^29^ For example, at room temperature, the addition of HO• to the double bond of alpha-pinene should not be concerned with ring opening. The requirement for accurate thermochemical and kinetic creates a major hurdle for applications involving complex organic structures. Additionally, RMG development has so far been focused on processes involving simple reactants with just a single organic functional group and up to one heteroatom: CH_4_, CH_3_CH_3_, CH_3_CH_2_CH_3_, exo-tetrahydrodicyclopentadiene, C_10_H_16_, CH_3_OCH_3_, CH_3_(CH_2_)_3_OH, CH_3_(CH_2_)_5_CH_3_, ((CH_3_)_2_CH)_2_CO, CH=CHCH=CHCH_2_CH_3_, HCC(CH_2_)_4_CCH, C_6_H_5_(CH_2_)_5_CH_3_, (CH_3_)_2_CHCH_2_OH, CH_3_(CH_2_)_4_CH_3_, H_2_NCH_2_CH_3_, and ((CH_3_)_3_C)_2_S, C_6_H_5_OH. A few other examples of data sources containing elementary steps are the NIST Chemical Kinetics Database,^36^ Mechanism and Catalytic Site Atlas (M-CSA),^37^ and Master Chemical Mechanism,^30,38−43^ all of which suffer from an unorganized, unstructured form of elementary steps with extremely limited online support.

## RMechDB: Underlying Data Set

### A Data Set of PLAUSIBLE Radical Elementary Steps

Organic transformations in databases such as REAXYS, SciFinder, and ORD are easily validated because published products are rigorously characterized using convenient spectroscopic techniques such as mass spectrometry, NMR, and IR. In contrast, mechanistic steps with one transition state are not easily validated. Experimental proof of a mechanistic step usually requires electronic structure calculations and/or laborious experimental tools such as chemical kinetics, isotopic labeling, crossover experiments, etc. It is often quoted that one can never prove a mechanism but only disprove the plausible alternatives.^44^ We set out to construct a data set of plausible elementary reaction steps, which are useful to chemists in constructing mechanistic pathways and predicting byproducts of organic reactions. Plausibility is subjective. For RMechDB, we define an elementary mechanistic step as plausible if a half-life of a day or less is expected at room temperature under the conditions cited. If more than one pathway has been postulated in the literature, it is expedient to include steps from both potential pathways in the data set until the discrepancy is resolved. That way, any pathway proposed using the data will reflect the ambiguity in the body of literature. In theory, the plausibility of any elementary reaction step can ultimately be validated using electronic structure calculations.

### Composition of the RMechDB Data Set

The initial data set in RMechDB consists of over 5,300 pedagogically chosen elementary radical mechanistic steps based on published transformations. The majority of the published mechanistic steps had to be further decomposed into elementary reaction steps with individual transition states. Over 880 steps were taken from eight introductory^45−52^ organic chemistry textbooks, advanced organic chemistry books,^53,54^ and an atmospheric chemistry textbook.^55^ Over 800 reactions were taken from the primary research literature including mechanisms for common synthetic transformations (atom transfer, tin chemistry, radical cyclizations), autoxidation, atmospheric reactions, and explosives. The literature mechanisms also included steps leading to 14 common industrial polymers: ethylene, propylene, butadiene, chloroprene, isoprene, acrylamide, acrylic acid, methyl acrylate, ethyl acrylate, butyl acrylate, methyl methacrylate, acrylonitrile, styrene, *p*-methylstyrene, vinyl chloride, vinyl fluoride, tetrafluoroethylene, chlorotrifluoroethylene, vinylidene fluoride, vinyl acetate, *N*-vinylpyrrolidinone. The conditions for polymerization, often including more than one type of initiator, were taken from the research literature and are not necessarily the proprietary initiators and conditions used for industrial synthesis. The data from textbooks and research literature are considered the core of the RMechDB database.

The core data set has been augmented with a large number of mechanistic steps related to the atmospheric oxidation of organic molecules. We refer to this data set as specific steps. A large number (847) of specific steps were taken from a comprehensive review of atmospheric isoprene oxidation that traced the fate of each individual carbon atom detailing the highly branched pathways from reaction with HO•, O_2_, NO, Cl•, and other species.^56^ For simplicity, we focus on the daytime atmospheric chemistry of isoprene at atmospherically relevant conditions (average atmospheric *T* = 278 K), neglecting elementary steps involving NO_3_, which is a dominant nighttime oxidant. Most of the elementary steps were inferred from composite transformations. About 3,000 mechanistic steps were coded from the first two stages of the major oxidation pathways in the Master Chemical Mechanism (MCM).^30^ The MCM contains mechanisms for atmospheric oxidation of 143 volatile organic compounds initiated by both HO• and NO_3_, including reactions of isoprene. Steps more than ten times slower than the fastest process (with the same reactants) were also excluded. Steps second-order in reactive intermediates were excluded on the assumption that they would not slow under typical conditions. For both the Wennberg and MCM steps, transformations initiated by pericyclic [3 + 2] cycloaddition of O_3_ with alkenes were excluded from this initial data set, but depicting the cycloaddition as a diradical process could be an expedient.^57^ Photolysis steps were also excluded. Any steps left out of this initial data set can be introduced in the future.

**Figure 2:**
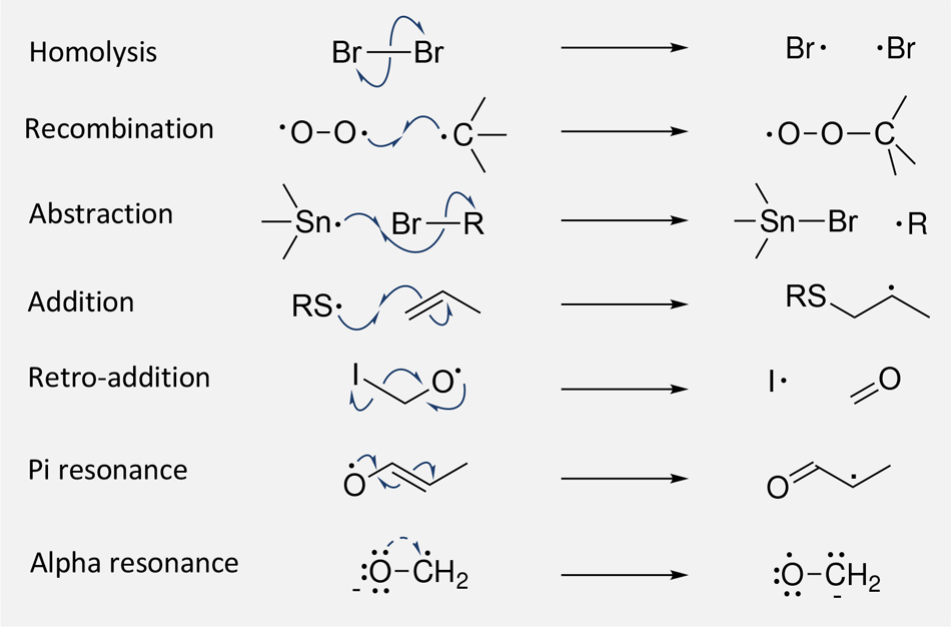
Seven different categories of mechanistic steps involving radicals.

**Figure 3:**
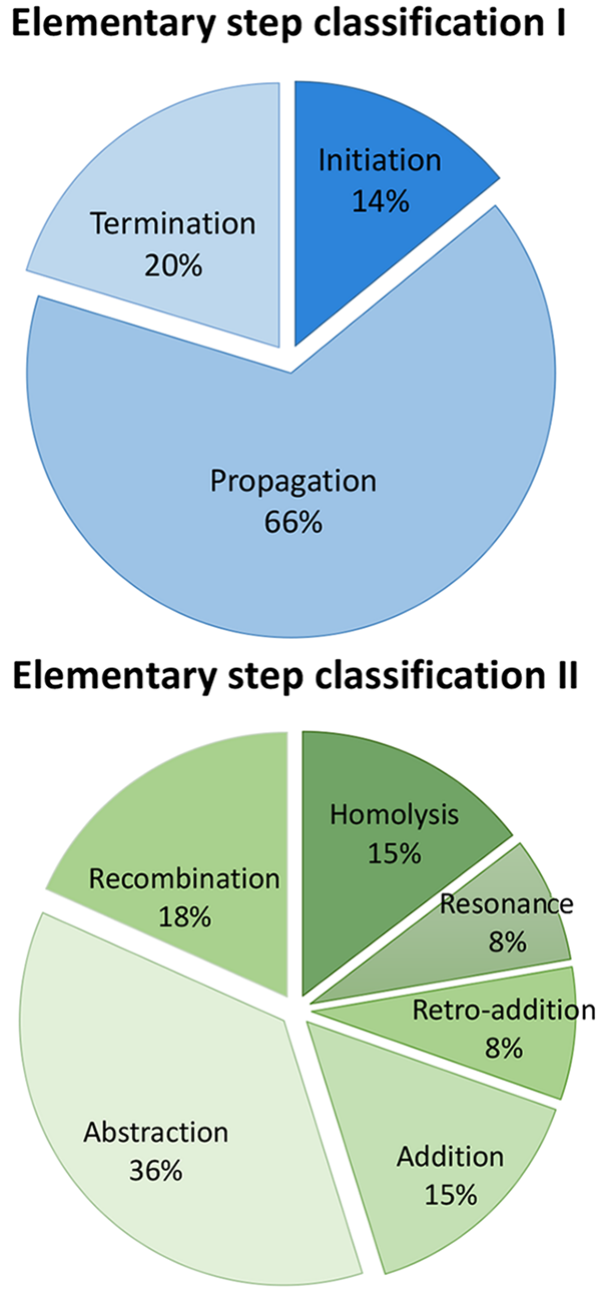
Distribution of the different classes of reaction in the current version of the RMechDB data set.

The individual mechanistic steps are also labeled using two distinct classification schemes: (1) three-class classification, where each elementary step falls into one of the three possible phases of a radical chain reaction: initiation, propagation, and termination, and (2) the more detailed seven-class classification, where an elementary step reaction falls into one of seven different categories: homolysis, recombination, abstraction, addition to pi bonds, retro-addition to pi bonds, and pi (e.g., allylic) and alpha lone pair resonance (e.g., ketyls). All seven classes are depicted in Figure 2. In RMechDB, resonance is represented as a mechanistic step, even though there is no transition state. Homolysis and recombination are mechanistic reverses of each other, like addition and retro-addition. Alpha resonance is represented with a single curved half arrow, but it is acknowledged that the half arrow falsely implies the formation of a partial double bond. The steps in radical chain mechanisms are often classified as initiation, propagation, or termination steps, but many transformations involving radicals do not involve chain mechanisms. Homolysis is a typical chain initiation step. Atom abstraction, addition, retro-addition, and resonance are typical chain propagation steps. Recombination is a typical chain termination step. Within the RMechDB data set, we try to emulate the natural distribution of radical reactions based on the classifications described above. Figure 3 represents the distribution of different classes of radical reactions in the RMechDb data set.

### Structure of the Data

**Figure 4:**
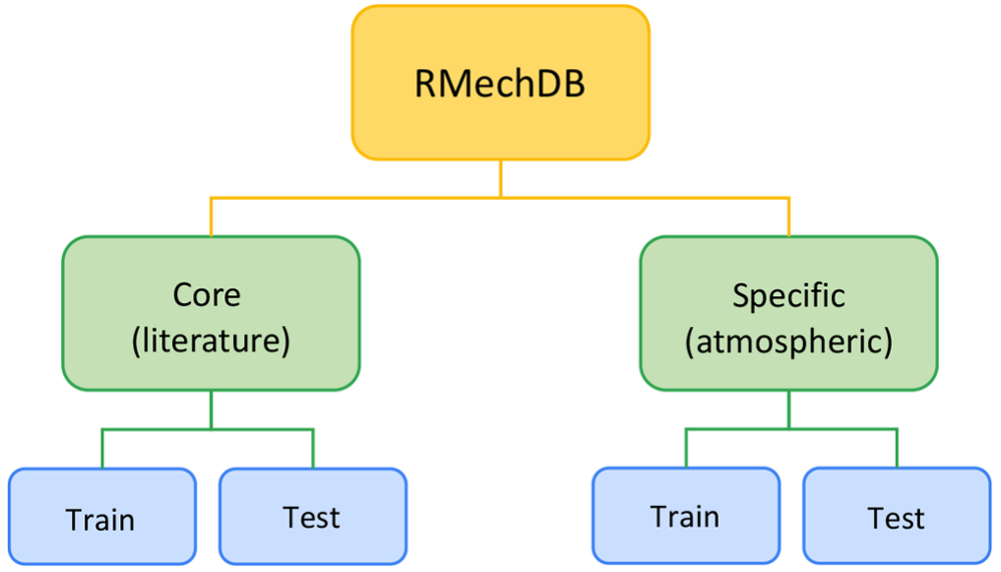
General format of the RMechDB data set.

The initial version of RMechDB contains over 5300 pedagogically chosen elementary radical step reactions based on published transformations. Steps are categorized into two major types: (1) core elementary steps, extracted and curated from textbooks and the scientific literature, capturing generic radical mechanisms, and (2) specific elementary steps, curated from multiple sources, capturing mechanisms associated with atmospheric chemistry. Given that one of the main goals for RMechDB is to provide a source of data for machine learning models, each type is carefully split into a canonical train and test data (Figure 4).

While machine-learning users can of course split the data in any way they want, having a canonical train/test data split facilitates standardized training and evaluation workflows, as well as the comparison of performance across different research groups. This canonical split is manually curated to ensure balance and coverage consistency between the train and test data. Specifically, we use two criteria: **balanced categorical distribution** and **consistent chemistry coverage**. To maintain the balance in categorical distribution, we ensure that the distribution of the seven categories described above (Figure 2) is approximately the same in the train and test data. To maintain consistent chemistry coverage, for any mechanistic steps in the train data, we ensure that there is at least one mechanistic step with similar reacting functional groups in the test data. As a result, using this presented train and test split leads to a more interpretable evaluation of the generalization capabilities of predictive models.

**Figure 5:**
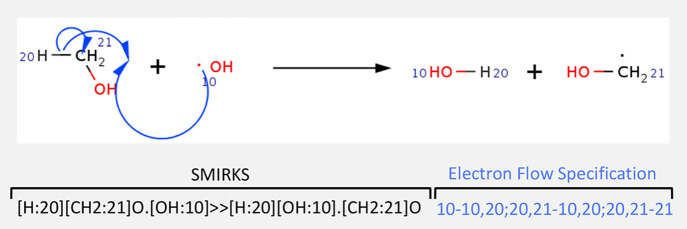
RMechDB format for depicting reactions and arrow-pushing mechanisms. The atoms participating in the reaction are mapped on both sides of the reaction.

Each entry of RMechDB consists of elementary reaction steps in the SMIRKS format including atom mapping for atoms that are a part of the transformation. Each SMIRKS is associated with its electron flow specification representing the atom indices on the curved half arrows (Figure 5). Additionally, each elementary step has been decorated with the following properties: (1) the initial condition of the reaction which falls into the room temperature (298 K), heat, or light conditions; (2) reaction class I which is the type of the radical elementary step; (3) reaction class II which is the type of the radical elementary step based on a more fine-grained categorization; and (4) the scholarly source of the elementary step. The addition of more important properties such as phase, solvent, wavelength, and enthalpy is left for future work.

## Standard Elementary Step Model

In addition to serving as a central source of reaction data for machine learning models, RMechDB is designed to be extendable by community contribution. To maintain that, it is crucial to use a standard and unified representation of elementary step reactions. This standard representation would enable consistent data sharing, model reproduction, and scalable expansion. We model the elementary step reaction using the reaction model introduced in refs (25 and 31). In this model–the so-called “elementary step model”, the transition state is modeled as the movement of one single electron from one-half-occupied molecular orbital (MO) to another. We use the atom labels in the arrow code of the elementary step to track the movement of the electron. Lone pairs or π-bonds adjacent to π-bond MOs can be chained to allow longer-range resonance rearrangement. In this model, each MO is associated with its main atom. As a result, each radical elementary step has two reactive atoms and two reactive MOs. We use the elementary step model to construct and populate the database schema described in the next section.

## RMechDB: The Core Database

### Database Schema

The database is implemented using the PostgreSQL^58^ database management system,^59^ to store, query, and retrieve reaction instances both efficiently and safely. We use OpenEye Scientific Software^60^ toolkits OEChem,^61^ OEDepict,^62^ and GraphSim^63^ for chemoinformatics processing and depiction. In addition, we use Chemaxon Marvin^64^ for displaying and characterizing chemical structures, substructures, and steps with their corresponding arrow-pushing mechanisms.

**Figure 6:**
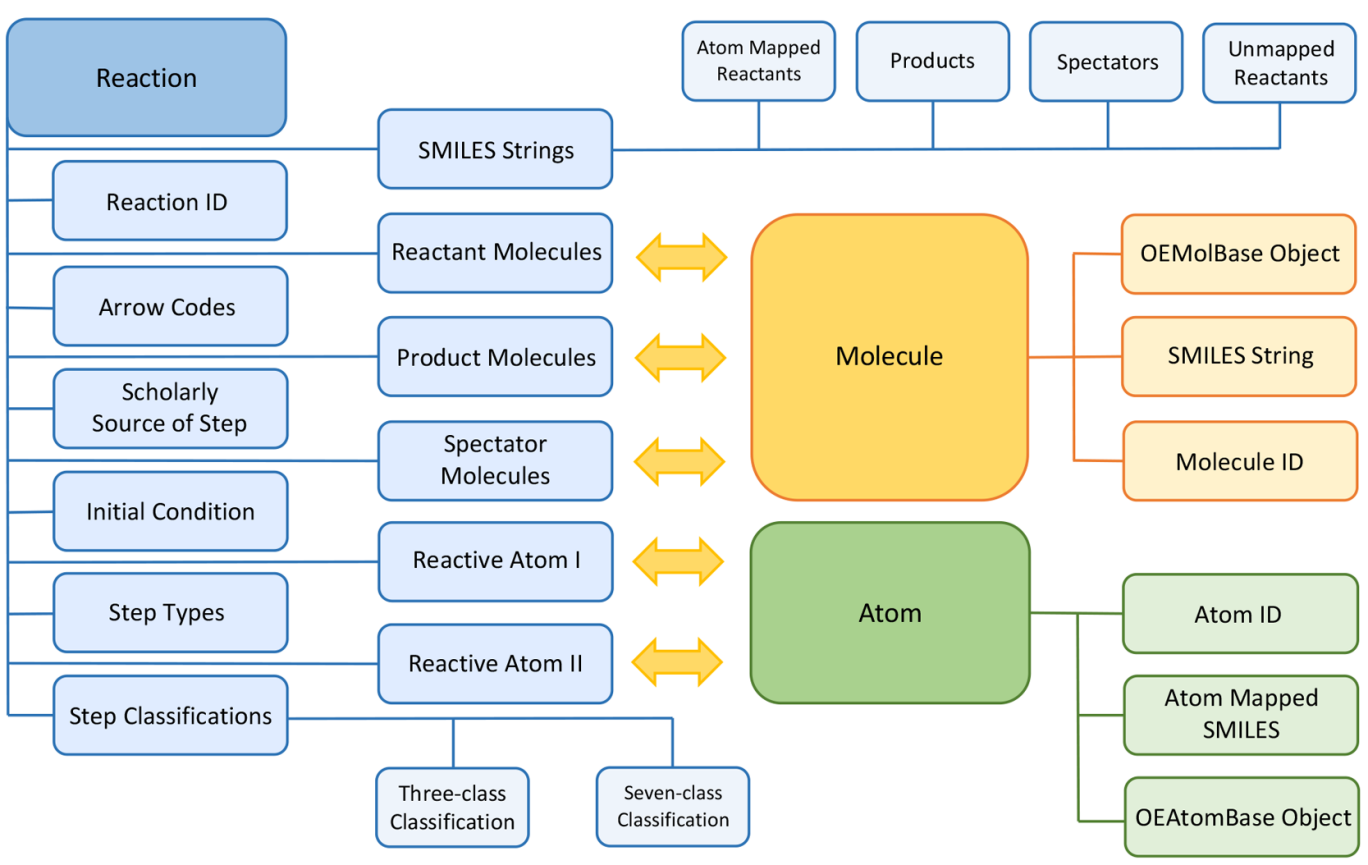
Three fundamental models of the RMechDB database and how they integrate. The yellow arrows show the many-to-many relations.

The RMechDB database schema comprises three fundamental models: (1) Reaction, (2) Molecule, and (3) Atom, as shown in Figure 6. The inter- and intraintegration of these three models allow for fast and efficient reaction search and retrieval. As the naming suggests, each elementary step is stored as an instance of the Reaction model which comes with several descriptive fields. These fields are designed to uniquely represent an elementary step reaction and all the available metadata associated with it. Here, we list the main fields of the Reaction model.

1. **Reaction ID:** Each reaction is associated with a unique ID number.
2. **Canonicalized atom mapped SMILES of the reactants:** The SMILES string of the reactants’ molecules, with integer labels for atoms that are participating in the reaction. We use a labeling convention where the labels of the participating atoms on the nucleophile part start from 10 and increment by one per atom and the labels of the participating atoms on the electrophile part start from 20 and increment by one per atom.
3. **Canonicalized SMILES of the products:** The unique SMILES representation of the product molecules generated from the reactive reactants.
4. **Canonicalized arrow codes:** The standard codes for arrow-pushing mechanisms contain the integer labels of the participating atoms on the reactants’ side. The standard arrow codes begin from the integer label (starting at 10) on the nucleophilic group.
5. **Spectator molecules:** The unique SMILES representation of the molecules that are present in the reaction but not participating in the electron transfer.
6. **Reactive atom I:** The SMILES string of the molecule containing the first reactive atom (based on the RMechDB orbital model) whose label is 1.
7. **Reactive atom II:** The SMILES string of the molecule containing the second reactive atom whose label is 1.
8. **Step type:** Core or specific step (Figure 4).
9. **Initial heat or energy:** The initial condition of the step which can be independent of external energy–represented as blank, “heat”, or “light”.
10. **Step classification I:** The class of the step according to the 3-class classification into initiation, propagation, and termination.
11. **Step classification II:** The class of the step according to the 7-class classification into homolysis, recombination, addition, retro-addition, abstraction, alpha resonance, and pi resonance shown in Figure 2.

Given the fields above associated with the Reaction model, an instance of the Reaction model in RMechDB can be uniquely retrieved from the database using either the **Reaction ID** or the combined properties 2–5 as the key.

The Molecule model has three fields corresponding to the unique molecule ID, canonicalized SMILES string of the molecule, and the OEChem MolBase object.^61^ An instance of the Molecule model has a many-to-many relation with the reactant molecules’, product molecules’, and spectator molecules’ fields of the Reaction model.

The Atom model has three fields corresponding to the unique ID, canonicalized atom mapped SMILES string of the parent molecule, and the OEChem AtomBase object.^61^ An instance of the Atom model has a many-to-many relation with the reactive atom I and reactive atom II fields of the Reaction model.

The schema with the fields described above is designed not only to provide efficient storage and retrieval but also to enable the automated population of the fields for new steps that are contributed to RMechDB by the community as described in the section on Uploading New Data.

## RMechDB: Web Server

The web server of the RMechDB includes three interfaces for (1) searching the data; (2) downloading the data; and (3) uploading new data.

### Searching the Data

RMechDB provides an interactive search interface available at https://deeprxn.ics.uci.edu/rmechdb/rsearch where users can search through the database using a variety of methods. At the highest level, the interface allows for reaction search and compound search.

#### Reaction Search

1. **Exact search:** Using the exact search method, the user inputs the query in the form of the SMIRKS of an elementary step containing reactants and products (no arrow code needed). Then the systems finds and displays all the elementary steps with the same reactants and products as in the query reaction but with additional molecules involved as reagents or spectators.
2. **Search by reactants:** Using the search by reactant (or by reactants), the user inputs the query in the form of a set of molecules, separated by a “.”. Upon hitting the search button, the system finds and displays all the elementary steps with reactants containing the query molecules. This search is useful when the user does not know the exact reaction and how molecular orbitals might react.
3. **Search by products:** Similar to the search by reactants, using the search by product (or by products), the user inputs the query in the form of a set of molecules, separated by a “.”. Upon hitting the search button, the system finds and displays all the elementary steps with products containing the query molecules.
4. **Similarity search:** Using the similarity search method, the user again inputs the query in the form of the SMIRKS of an elementary step containing reactants and products (no arrow code needed). Then the user specifies a similarity metric and the number of similar reactions (*N*) to be retrieved under this query. Upon hitting the search button, *N* elementary steps sorted from the most similar to the least similar to the input query are displayed.

The current version of RMechDB is equipped with the following similarity metrics computed on various representations of the elementary steps:

1. The Tanimoto, dice, and cosine distance between the binary Extended Connectivity Fingerprints (ECFP) of the elementary steps.
2. The Euclidean distance between the embedding of the elementary steps derived using a pretrained transformer architecture, trained on the SMIRKS of the USPTO data set.^17,21^
3. The Euclidean distance between the embedding of the elementary steps derived using the pretrained RxnHypergraph method.^11^

#### Compound Search

In addition to search capabilities based on elementary steps, RMechDB provides search capabilities based on smaller chemical entities as follows:

1. **Molecule search:** In this search, the user inputs the SMILES string of the desired molecule. After testing the validity of the input SMILES, RMechDB displays those elementary steps in the database that contains the desired molecule in the reactant or product side of the elementary step.
2. **Reactive atom (molecular orbital) search:** In this search, the user inputs the atom-mapped SMILES string of the molecule where the reactive atom is labeled using an integer between 1 and 9, while the other atoms are not labeled. After testing the validity of the input SMILES with the labeled atom, RMechDB displays all the elementary steps in the database where the labeled atom is acting as one of the two main reactive atoms in the elementary step.
3. **Substructure search:** In this search, the user inputs the SMARTS of a chemically valid substructure. RMechDB displays all the elementary steps in the database with molecule(s) containing the input substructure. The molecule that contains the input substructure can be in the reactant or product side of the elementary step.

In addition, the results of each search can also be filtered using the following properties: (1) the type of the elementary steps (core or atmospheric) and (2) the category of the elementary step based on either of the two categorization schemes described in the Composition of the RMechDB Data section.

The result of each search will be displayed as a table containing the depiction of the filtered reactions along with their reactive atom-mapped SMIRKS, arrow codes, masses of the products, and the initial conditions. The search query inserted by the users will also be displayed in a separate box.

### Downloading the Data

The data set of the chemical reactions in RMechDB is available for download at https://deeprxn.ics.uci.edu/rmechdb/download. The data set is licensed under the *Creative Commons Attribution-NonCommercial-NoDerivs (CC-BY-NC-ND)* license, which limits its free public usage to noncommercial purposes. Under this license, the users are not allowed to modify and distribute the data set or to distribute the original data set without referencing the original source. After submitting basic information (name, email, and institution) and accepting the license terms, users receive an email containing a comma-separated value (CSV) file containing all the data and metadata.

### Uploading New Data

While we continue to insert new data in RMechDB, we invite the community to contribute new radical elementary steps. Uploading new data can be done at https://deeprxn.ics.uci.edu/rmechdb/upload.

**Figure 7:**
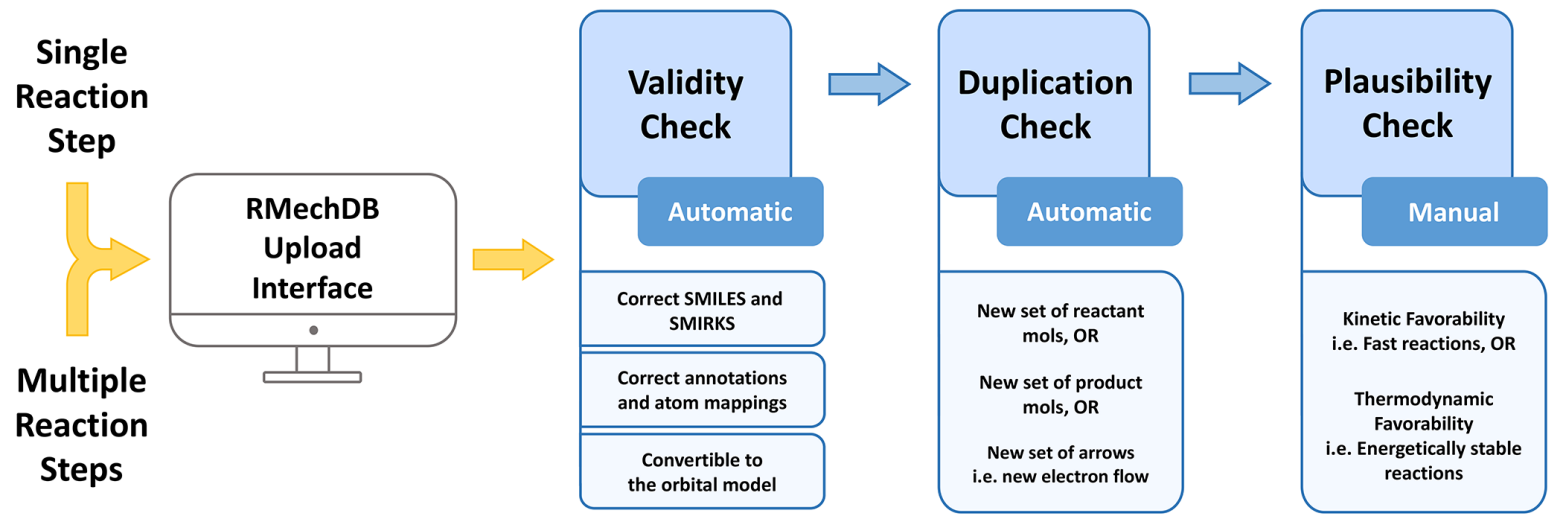
Schematic depiction of how new data contributed to RMechDB and goes through different checking stages.

Contributing users must fill out two fields: (1) the SMIRKS of the elementary step and (2) the corresponding electron flow specification (codes for arrow pushing), as shown in Figure 5. There are also two optional fields where the user can provide information about the source of the elementary step (e.g., the title of a textbook, or a publication) and provide an optional note (e.g., the necessity of initial energy). After uploading the elementary step, it will be checked for validity, duplication, and plausibility (Figure 7).

#### Validity Check

A submitted elementary step is considered to be valid if it satisfies the following three criteria:

1. The SMILES string of all the molecules on both sides of the submitted elementary step must be correct and convertible to graphs representing valid molecules. We use the Openeye Scientific Software^60^ toolkit OEChem^61^ to convert the input SMILES/SMARTS strings into molecular graphs.
2. The annotations for the arrow-pushing mechanisms must be correct. This implies that the reacting atoms on the reactant side of the elementary step must be labeled with distinct integers. These integers form the basis for the arrow-pushing mechanisms associated with electron transfers. The arrow codes must be consistent with the integers used to label the reacting atoms. An example of a valid atom mapping and arrow codes is shown in Figure 5.
3. The entered SMIRKS and arrow codes are then used to extract the interacting orbitals. We used our elementary step model described in the Standard Elementary Step Model section to create the elementary step object. Using this object, we extract the interacting molecular orbitals and their corresponding atoms. If the input SMIRKS and arrow codes fail to create the elementary step object, the input is considered invalid. This failure usually implies a mismatch between the labeled atoms and the corresponding arrow codes.

#### Duplication Check

In this step, we check that the valid uploaded elementary step is not equivalent to any elementary step already included in the RMechDB data set. We consider two steps to be equivalent if they have the same:

1. Canonicalized SMILES string of the reacting molecules.
2. Canonicalized SMILES string of the product molecules.
3. Canonicalized SMILES string of the spectator molecules.
4. Conventional representation of the codes for the arrow-pushing mechanism. The labels of the participating atoms on the nucleophilic component start from 10 with increments of one per atom, and the labels of the participating atoms on the electrophilic component start from 20 with increments of one per atom. It is important to mention that the user can use any integers to label the participating atoms. The conventional arrow codes will be automatically generated by RMechDB.

Once an elementary step is uploaded, RMechDB performs the validity and duplication tests automatically. In case of failure of either test, an informative error message is displayed with details about the corresponding errors.

#### Plausibility Check

Once the submitted elementary step passes both tests, it is further manually reviewed by the RMechDB curators for overall quality and plausibility, before being imported into the RMechDB.

## Conclusion

The main obstacle for the large-scale application of AI methods to chemical reactions is the lack of data.^20^ Some efforts have begun to try to address this fundamental bottleneck at the level of chemical transformations.^21,32^ Here, we have presented a complementary effort aimed at building an open platform and database, RMechDB, for elementary steps in radical reactions. A parallel effort is underway to cover also polar reactions.

Databases of elementary steps introduce a new perspective and new opportunities for computer-aided reaction prediction and modeling. In particular, when properly deployed, they should facilitate addressing the central problems of explainability and causality found in many applications of AI in chemistry and other domains. The ability to decompose a transformation into a sequence of elementary steps is one way to understand how and why it occurs.

The RMechDB platform is designed to facilitate training deep learning and other AI models in data-driven workflows using its tabular data, with no need for additional preprocessing steps. While RMechDB is designed primarily to facilitate the training and evaluation of data-driven models for predicting all the potential outcomes of radical reactions, it can be used also for other tasks, such as reagent versus reactant classification, initial condition prediction, and reaction classification.

RMechDB is intended to be a live platform for contributing, aggregating, curating, and distributing data in the form of elementary radical reaction steps to accelerate research in chemoinformatics and reaction modeling. It provides a unified model that ought to facilitate data sharing, model building, dissemination, and publications. Future updates will be reported through the RMechDB Web site at https://deeprxn.ics.uci.edu/rmechdb. We encourage the community to explore and use the RMechDB data and functionalities and contribute to its expansion.

## Data and Software Availability

The RMechDB Web site is accessible at https://deeprxn.ics.uci.edu/rmechdb. The RMechDB data set can be downloaded through the download interface at https://deeprxn.ics.uci.edu/rmechdb/download. Documentation on how to use the RMechDB interfaces is also provided at https://deeprxn.ics.uci.edu/rmechdb/howtouse.

## Supporting Information Available

RMechDB (online platform for aggregating, curating, and sharing radical reaction data in the form of elementary step reactions): instructions and examples on how to use three interfaces for searching, downloading, and uploading data (PDF)
