# Supplementary material for: RMechDB: A Public Database of Elementary Radical Reaction Steps
Source: J Chem Inf Model. 2023 Feb 17;63(4):1114–23. doi: 10.1021/acs.jcim.2c01359 (PMC9976277; doi:10.1021/acs.jcim.2c01359)

RMechDB: A Public Database of Elementary Radical Reaction Steps

Mohammadamin Tavakoli,[†,¶] Yin Ting T. Chiu,[‡,¶] Pierre Baldi,[*,†] Ann Marie Carlton,[‡] and David Van Vranken[*,‡]

†*Department of Computer Science, University of California, Irvine, Irvine, California 92697, United States*

‡*Department of Chemistry, University of California, Irvine, Irvine, California 92697, United States*

¶*Equal Contribution*

E-mail: pfbaldi@uci.edu; david.vv@uci.edu

Supporting Information

RMechDB is an online platform for aggregating, curating, and sharing radical reaction data in the form of elementary step reactions. The platform offers three interfaces for searching, downloading, and uploading data. In this document, we provide instructions and examples on how to use these three interfaces.

Searching the Data

The search interface allows for two categories of search: reaction search and compound search. The search interface is accessible at `http://deeprxn.ics.uci.edu/rmechdb/rsearch`. On this page, the user will find a set of input fields and other parameters. Here we explain how to fill out the query field and choose the corresponding search type. The rest of the parameters are

self-explanatory. The user can also refer to the main manuscript for more information.

Reaction Search

The Reaction Search is accessible at `http://deeprxn.ics.uci.edu/rmechdb/rsearch`. The user needs to make sure that the blue button for *Search by reaction* is activated. The user can toggle between the following search types using the key for *Choose a search type*.

Exact search Using the exact search method, the user inputs the query in the form of the SMIRKS of an elementary step containing reactants and products. Then the system finds and displays all the elementary steps with the same reactants and products as in the query reaction possibly with additional molecules involved as reagents or spectators. Here are two examples of the query and the corresponding search results:

As one can understand from the acceptable SMIRKS, the order of molecules within the reactants and products would not affect the search results. Different atom mappings would not affect the search results. There is no need to input arrow codes along with the reaction for this type of search. If the user inputs arrow codes, they will be discarded.

Search by reactants Using the search by reactant, the user inputs the query in the form of a set of molecules, separated by ".". Upon hitting the search button, the system finds and displays all the elementary steps with reactants containing the query molecules. This search is useful when the user does not know the exact reaction and how molecular orbitals might react.

Search by products Similar to the search by reactants, using the search by products, the user inputs the query in the form of a set of molecules, separated by ".". Upon hitting the search button, the system finds and displays all the elementary steps with products containing the query molecules. Examples are provided in Table 2.

Table 1: Examples of the exact search.

<table>
<tr><td>Acceptable Query</td><td>C1=CC=C(C=C1)CC[O:10][N+:11](=O)[O−]>>c1ccc(cc1)CC[O].[N+](=O)[O−]
C1=CC=C(C=C1)CC[O:10][N+:11](=O)[O−]>>[N+](=O)[O−].c1ccc(cc1)CC[O]
C1=CC=C(C=C1)CC[O:24][N+](=O)[O−]>>c1ccc(cc1)CC[O:19].[N+:4](=O)[O−]</td></tr>
<tr><td>Query Depiction</td><td>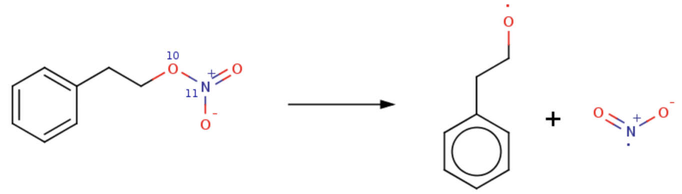</td></tr>
<tr><td>Results Depiction</td><td>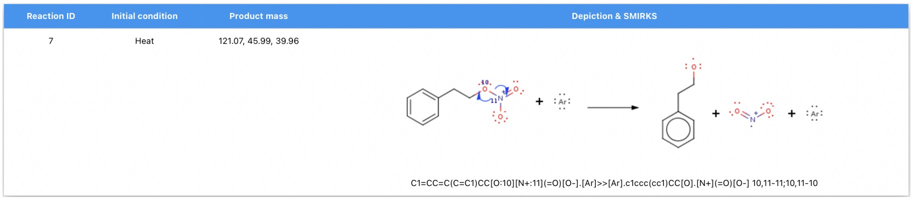<table><tr><th>Reaction ID</th><th>Initial condition</th><th>Product mass</th><th>Depiction & SMIRKS</th></tr><tr><td>7</td><td>Heat</td><td>121.07, 45.99, 39.96</td><td>C1=CC=C(C=C1)CC[O:10][N+:11](=O)[O-].[Ar]>>[Ar].c1ccc(cc1)CC[O].[N+](=O)[O-] 10,11-11;10,11-10</td></tr></table></td></tr>
<tr><td>Acceptable Query</td><td>[CH2:20]C1=CC=CC=C1.[N+:10](=O)[O−]>>c1ccc(cc1)C[N+](=O)[O−]
[CH2:3]C1=CC=CC=C1.[N+](=O)[O−]>>c1ccc(cc1)C[N+:9](=O)[O−:13]
[N+:10](=O)[O−].[CH2:20]C1=CC=CC=C1>>c1ccc(cc1)C[N+](=O)[O−]</td></tr>
<tr><td>Query Depiction</td><td>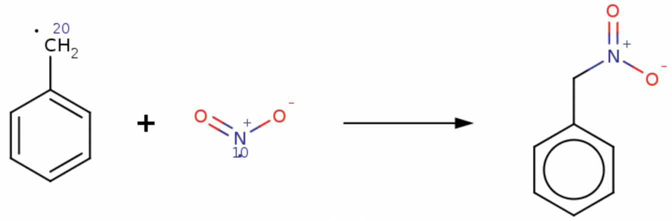</td></tr>
<tr><td>Results Depiction</td><td>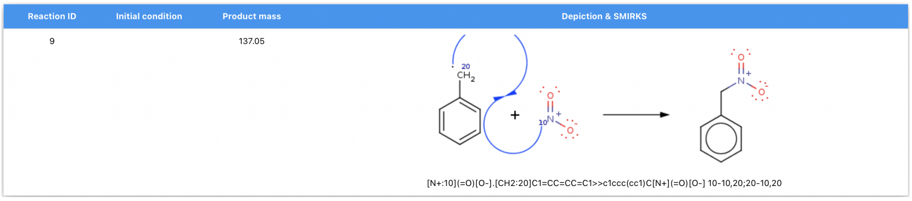<table><tr><th>Reaction ID</th><th>Initial condition</th><th>Product mass</th><th>Depiction & SMIRKS</th></tr><tr><td>9</td><td></td><td>137.05</td><td>[N+:10](=O)[O-].[CH2:20]C1=CC=CC=C1>>c1ccc(cc1)C[N+](=O)[O-] 10-10,20;20-10,20</td></tr></table></td></tr>
</table>

Similarity search The user must input a valid reaction in the form of the SMIRKS of an elementary step containing reactants and products (no arrow code needed) similar to the Exact Search method. Then the user specifies a similarity metric and the number of similar reactions. Depending on the selected similarity method, the most similar reactions to the input query will be displayed. Table 3 shows examples of valid input.

Table 2: Examples of search by reactants/products.

Acceptable Query	C[C](C)C.[N+](=O)[O−]\[N+](=O)[O−].C[C](C)C\C[C](C)C.[N+:9](=O)[O−:4]
Query Depiction	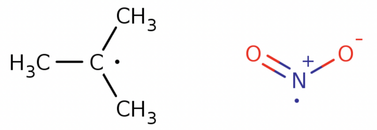
Results Depiction	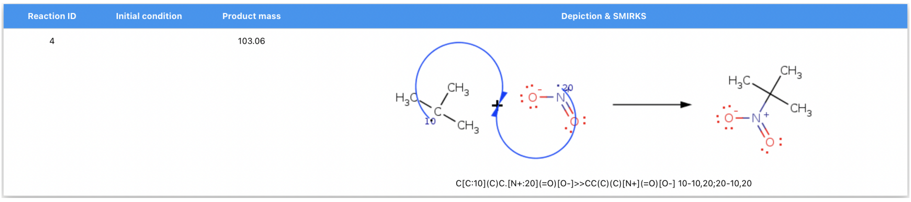
Acceptable Query	C[C](C)C.C=O\C=O.C[C](C)C\C[C](C)C.C=[O:19]
Query Depiction	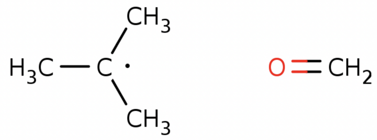
Results Depiction	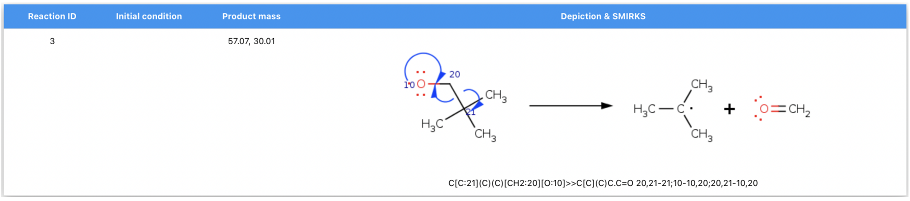

Compound Search

In addition to search capabilities based on elementary steps, RMechDB provides search capabilities based on smaller chemical entities including molecules, atoms, and substructures. The Compound Search is accessible at `http://deeprxn.ics.uci.edu/rmechdb/csearch`. The user needs to make sure that the blue button for *Search by compound* is activated. First, the user needs to select the compound type using the drop-down item at the top of the page. Depending on the selected compound, the user can input the proper search query described below:

Molecule search In this search, the user inputs the SMILES string of the desired molecule. After testing the validity of the input SMILES, RMechDB displays those elementary steps in the database that contains the desired molecule in the either reactant or product side of the elementary steps. The atom mapping and the canonicalization of the input SMILES string would not affect the search results. Table 4 shows an example of the compound search where the selected compound is a

Table 3: Examples of the inputs for the similarity search.

<table>
<tr><td>Acceptable Query</td><td>CC1=CC=CC=C1.[N+](=O)[O−]>>c1ccc(cc1)C[N+](=O)[O−]
[N+](=O)[O−].CC1=CC=CC=C1>>c1ccc(cc1)C[N+](=O)[O−]
CC1=CC=CC=C1.[N+](=O)[O−]>>c1ccc(cc1)C[N+:20](=O)[O−:12]</td></tr>
<tr><td>Query Depiction</td><td>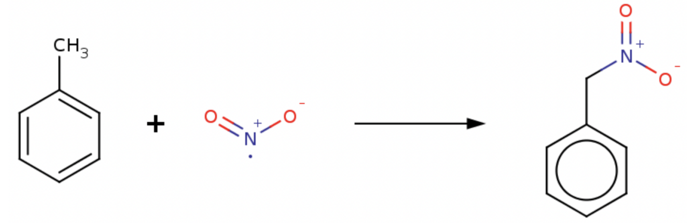
</td></tr>
<tr><td>Results Depiction</td><td>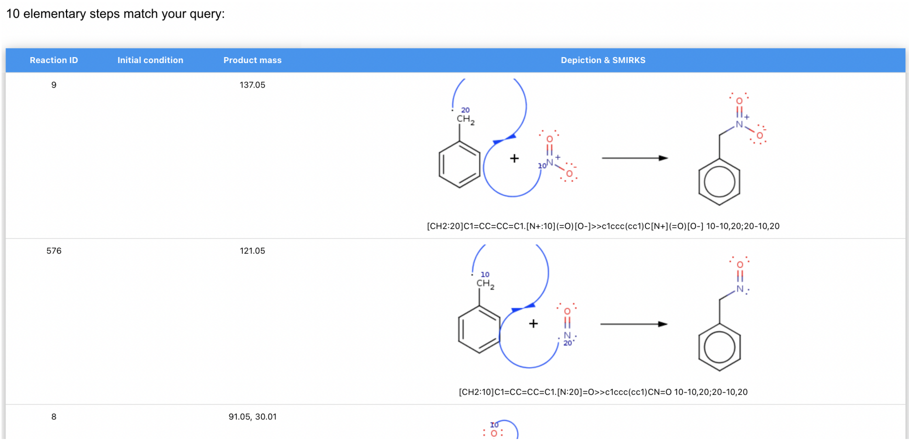
</td></tr>
</table>

molecule.

Reactive atom search In this search, the user inputs the atom-mapped SMILES string of the molecule where the reactive atom (the desired atom) is labeled using an integer between 1 and 9, while the other atoms are not labeled. After testing the validity of the input SMILES with the labeled atom, RMechDB displays all the elementary steps in the database where the labeled atom is acting as one of the two main reactive atoms in the elementary step. The canonicalization of the input SMILES string would not affect the search results. However, the atom mapping must be as described where only one atom is mapped and the rest are not. Table 5 shows an example of the compound search where the selected compound is an atom.

Substructure search In this search, the user inputs the SMARTS of a chemically valid substructure. RMechDB displays all the elementary steps in the database with molecule(s) containing the

Table 4: Examples of the proper inputs for the molecule search.

Acceptable Query	CC(C)(C)[N+](=O)[O−]\[CH:3]C(C)(C)[N+](=O)[O−:2]
Query Depiction	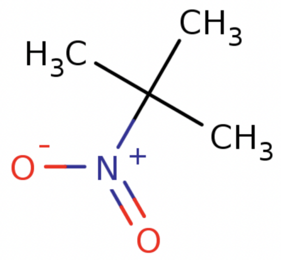
Results Depiction	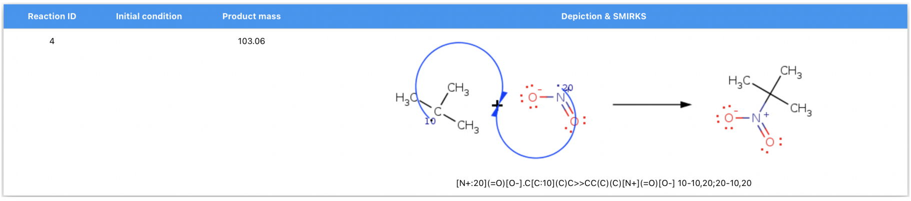

input substructure. The molecule that contains the input substructure can be in the reactant or product side of the elementary step. Table 6 shows an example of the compound search where the selected compound is substructure.

Downloading the Data

The download interface is accessible at `https://deeprxn.ics.uci.edu/rmechdb/download`. On this page, the user must enter his information and email address. Upon reading and agreeing to the terms of the CC-BY-NC-ND license, the user will receive an email containing the RMechDB data set. The RMechDB data set is a compressed directory with five comma-separated value (CSV) files:

1. *all.csv*: All the radical elementary steps in the RMechDB data set.

2. *train_core.csv*: All the core radical elementary steps chosen for training machine learning models.

3. *train_specific.csv*: All the specific radical elementary steps chosen for training machine learning models.

4. *test_core.csv*: All the core radical elementary steps chosen for testing machine learning models.

5. *test_scpecific.csv*: All the specific radical elementary steps chosen for testing machine learning models.

Each of the CSV files has five columns: (1) The SMIKRS of elementary steps; (2) The arrow codes of elementary steps; (3) The initial condition of elementary steps; (4) The category of elementary steps based on the three class classifications explained in the Composition of the RMechDB Data Set section of the RMechDB paper; (5) The category of the elementary step is based on the seven class classifications explained in the Composition of the RMechDB Data Set section of the RMechDB paper.

Uploading the Data

There are two methods to contribute to RMechDB by uploading the elementary step data: Upload a single step and upload multiple steps. Both methods are accessible through the upload interface at `https://deeprxn.ics.uci.edu/rmechdb/upload`. The user can select one method from the two buttons at the top of the page.

Upload Single Step To upload one single elementary step data, the user must fill out the four fields available at `https://deeprxn.ics.uci.edu/rmechdb/upload`. These fields are:

1. SMIRKS: The SMIRKS of the elementary step containing reactants and products with proper labels for the reactive atoms.

2. Arrows: The arrow codes represent the molecular orbital interactions. These arrows must match the label of the reactive atoms presented in the SMIRKS field.

3. Original source: This is an optional field where the user can provide the source from which the reaction was found/extracted.

4. Note: This is an optional field where the user can provide any auxiliary information about the reaction such as the initial condition.

Upload Multiple Steps To upload multiple elementary step data at once, the user must access the multi-step upload page at `https://deeprxn.ics.uci.edu/rmechdb/mupload` where he can upload a file containing the reactions. The uploaded file must be in the comma-separate value (CSV) format. Each row of the file must represent a reaction with four columns: (1) Reaction SMIRK; (2) Arrow code; (3) Original source; (4) Auxiliary information (e.g. initial energy, a special initial condition such as low pressure)

Table 5: Examples of the proper inputs for the reactive atom search.

<table>
<tr><td>Acceptable Query</td><td>CC(C)(C)[CH2:7][O]
CC(C)(C)[CH2:2][O]</td></tr>
<tr><td>Query Depiction</td><td>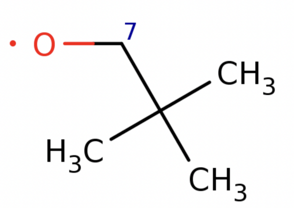
</td></tr>
<tr><td>Results Depiction</td><td>
<table>
<tr><th>Reaction ID</th><th>Initial condition</th><th>Product mass</th><th>Depiction & SMIRKS</th></tr>
<tr><td>3</td><td></td><td>57.07, 30.01</td><td>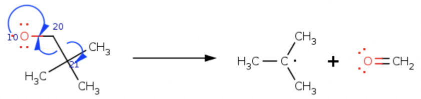

C[C:21](C)(C)[CH2:20][O:10]>>C=O.C[C](C)C 20,21-21;10-10,20;20,21-10,20</td></tr>
</table>
</td></tr>
<tr><td>Acceptable Query</td><td>C[C:9](C)C
C[C:1](C)C</td></tr>
<tr><td>Query Depiction</td><td>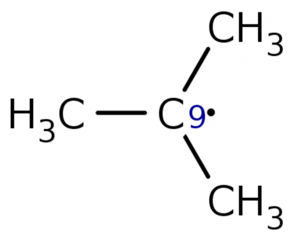
</td></tr>
<tr><td>Results Depiction</td><td>
8 elementary steps match your query:
<table>
<tr><th>Reaction ID</th><th>Initial condition</th><th>Product mass</th><th>Depiction & SMIRKS</th></tr>
<tr><td>4</td><td></td><td>103.06</td><td>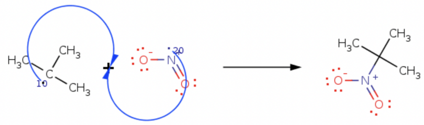

[N+:20](=O)[O-].C[C:10](C)C>>CC(C)(C)[N+](=O)[O-] 10-10,20;20-10,20</td></tr>
<tr><td>55</td><td></td><td>89.06</td><td>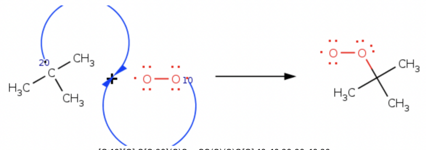

[O:10][O].C[C:20](C)C>>CC(C)(C)O[O] 10-10,20;20-10,20</td></tr>
<tr><td>346</td><td></td><td>92.04, 98.93</td><td></td></tr>
</table>
</td></tr>
</table>

Table 6: Examples of the proper inputs for the substructure search.

Acceptable Query	[CX4]

Results Depiction

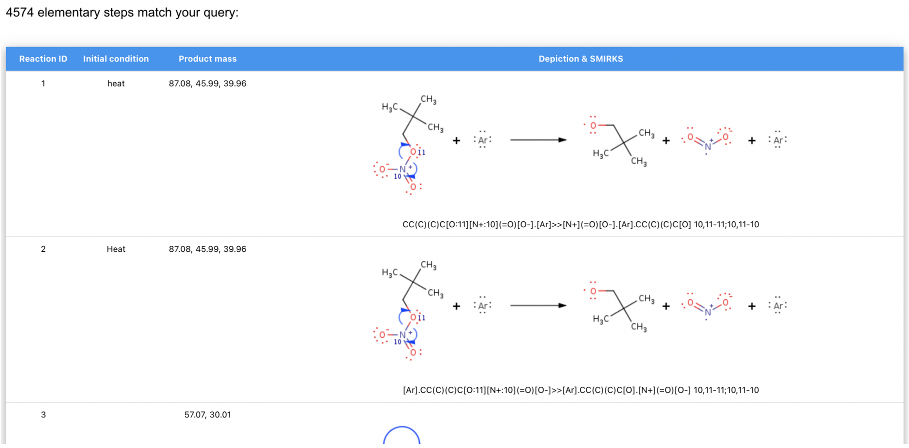

Acceptable Query	C1CCCCC1

Results Depiction

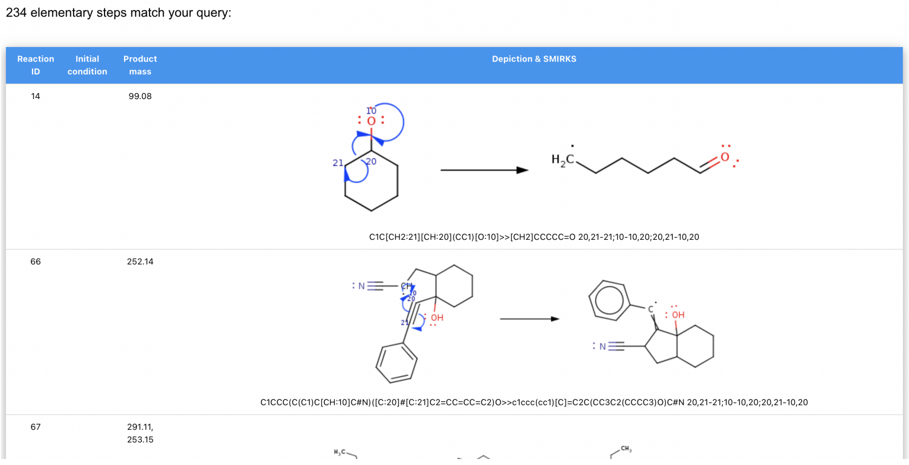

Supplement: Supplementary file 1 — ci2c01359_si_001.pdf [file ci2c01359_si_001.pdf]
